# Learning relationships in community-based service-learning: a social network analysis

**DOI:** 10.1186/s12909-019-1522-1

**Published:** 2019-04-25

**Authors:** Fabian P. Held, Chris Roberts, Michele Daly, Claire Brunero

**Affiliations:** 1 Office of the Deputy Vice-Chancellor (Education), University of Sydney, Sydney, Australia; 2 Faculty of Medicine and Health, University of Sydney, Sydney, Australia; 3 University of Sydney, Rural Clinical School (Broken Hill), Sydney, Australia; 4 University of Sydney, Rural Clinical School (Broken Hill), Broken Hill, Australia

**Keywords:** Social networks, Peer learning, Service-learning, Supervision, Clinical education, Allied health students

## Abstract

**Background:**

Little is known about the social learning of students within community-based clinical placements and ways in which it can be supported. In an allied health service-learning program, we analysed students’ learning relationships to quantify what, and from whom students learnt.

**Methods:**

We conducted a social learning network survey in four domains of learning (clinical knowledge, procedural skills, professional development, and complex determinants of health) to explore learning relationships (*ties*) with other people (*alters)* that students (*egos*) formed during their placement. We quantified how different roles (supervisors, health professionals, administrators, peers, schoolteachers, and clients) contributed to the students’ learning in each of the four domains. We used exponential random graph models (ERGMs) to test which relational processes contributed to the structure of the observed learning networks.

**Results:**

Data was available from a complete cohort of 10 students on placement in a network of 69 members, thus providing information on 680 potential learning relations. Students engaged in similar ways in the domains of clinical knowledge, procedural skills, and professional development. Learning relations with academic supervisors were significantly more likely. Also students reported reciprocal learning relations with peers – i.e. they formed learning pairs. This effect was absent in learning networks about complex determinants of health (including socio-economic and cultural factors). Instead, local administrative staff were significantly more often the source of learning about the local contextual factors.

**Conclusions:**

Understanding the structure of student learning networks through social network analysis helps identify targeted strategies to enhance learning in community-based service-learning programs. Our findings suggest students recognised important learning from each other and from administrative personnel that is unrelated to the content of their placement. Based on this insight clinical educators could prepare students to become agentic learners, learning with each other and from sources outside their program.

**Electronic supplementary material:**

The online version of this article (10.1186/s12909-019-1522-1) contains supplementary material, which is available to authorized users.

## Background

Clinical placements for allied health students are in short supply internationally, leading to the development and evaluation of a number of models to maintain learning and teaching capacity [1–5] One consequence has been the development of community–based service-learning models [3, 6, 7] reflecting a move away from relying on traditional hospital based placements. There is a growing literature describing various aspects of such placements, including program design and implementation, learning outcomes assessment, the effects of the learning environment [8, 9], involvement of the community [10–13], students’ learning strategies, supervisory arrangements, and student engagement with patients/clients as well as with their peers. Despite a growing understanding of the positive effects of community-based service-learning programs and the many factors that contribute to students’ learning, there has been little theoretically informed explanation of which aspects of these programs contribute to which aspects of learning. This gap in the research is problematic for educators in that without evidence of the nature of student learning in these types of placement, there is uncertainty of which elements should be supported from scare resources. In this study we use the novel methodology of social network analysis to explore important aspects of learning of allied health professional (AHP) students in a rural community-based program. Our goal is to provide a rich account of *who* students perceived they learned from and whether there were any differences in different domains of learning, broadening the evidence basis for supervisory arrangements, especially in non-traditional community-based placements.

Service-learning exposes students to a range of people in their workplace, and clinical educators are expected to create programs which align student experiences with what practicing health professionals are expected to know, do and value [14, 15]. Therefore learning can occur in a number of work sites, including on hospital wards, within treatment rooms, operating theatres, conference rooms, patient/client homes, offices, corridors, ward stations and even in staff tea-rooms [16]. There is an extensive literature on how students might be expected to learn through participating at work [17] and engage in ‘learning that is stimulated by workplace activities’ [18].

The effects on a variety of student learning outcomes have been reported across a range of domains, including improved intra- and interpersonal skills, academic knowledge and professional skills, and civic engagement as well as social responsibility. Specifically, students exhibited improved clinical skills and subject-specific knowledge [19–21], a broader understanding of the roles and responsibilities of health professionals [12, 22, 23], a more nuanced understanding of the complex determinants of health [11, 21, 23, 24], and that community-based placements had a significant part to play in rural career intention [25].

In rural community-based placements, students face particular barriers to workplace learning. A common barrier is limited access of students to existing AHPs, high workloads of these professionals, and limited resources to support the coordination of student learning experiences [26]. A further problem is supervisory capacity when facing increasing number of students undertaking clinical activities [27, 28]. In this context, some universities have invested in appointing rural clinician academics to undertake supervision in allied health [29].

Aiming to complement and improve supervision arrangements, developers of non-traditional practice education models investigated the extent to which students also learn from their peers [30, 31]. Peer learning can address specific gaps in delivering the curriculum [32] including providing additional student support in preparation for assessments [33], leadership, coaching, and learning skills opportunities, and can enhance both confidence and intrinsic motivation [27]. Peer learning can be a valuable method of enriching students’ learning experience and is thought to be an effective and efficient way to introduce and foster core professional skills that may not be included in formal health care professional curricula [30]. A collaborative model where one clinical educator supervises two or more students concurrently provides opportunities for peer learning, and enables more supervisory capacity [3]. At the same time, student engagement within a placement depends on both the learner and the context [14, 15, 34]. Program design may aim to create and promote opportunities for engagement inviting students to move away from passively following learning instructions typical of traditional placements [35].

### Theoretical framework

In providing a novel methodology to visualize and analyse student learning relationships in community-based clinical placements, we acknowledge a number of educational frameworks used in these contexts. They have the common aim of facilitating the transfer of theory into practice in the workplace through situated and experiential learning. These include for example practice based learning [36, 37], experiential learning [38], service-learning [39], work-based learning [17], peer learning [27, 32, 33], and integrated clinical placements [40]. Common features to all these models include the alignment of defined learning outcomes with clinically situated and supervised learning activities, with appropriate student support and authentic assessment.

Some of these frameworks draw on a rich literature on the social nature of learning in the workplace. For medical students, the social nature of learning appears to be a significant feature in rural integrated clinical placements [41] compared with traditional short-term hospital placements [42]. Social learning may include unstructured and unplanned interactions such as ad-hoc reflections with peers, or serendipitous conversations with administrators [43, 44]. Recent methodological innovations enable us to characterise the process of social learning in greater detail, mapping relationships within a group of people as a social network [45]. Such networks can be analysed quantitatively with specialised statistical methods [46]. In educational research, network approaches have captured the structure of interactions within an entire group including relationships that are part of the structured curriculum, as well as those outside [47, 48]. In the UK and the Netherlands, social network analysis suggested that medical students chose friends from their same gender and ethnic group [49] and that friendships were related to subsequent assessment performance, implying that students were learning with their friends [47].

Theories underpinning the study of relationships within networks draw on a rich base of literature. Processes that have been described as important to the formation of human relations include the tendency to reciprocate relations [50], and homophily, which describes the tendency to connect to people who share similar attributes [51]. Structural balance theory explains why people avoid making friends with the friends of an enemy [52]. Triadic closure is the tendency of connecting friends-of-friends, forming a triangle [53]. This effect has consequences for the transmission of information in a network, where densely connected groups of people form their own echo chamber, while receiving novel information through the few relations that reach out of these groups [54]. Lastly, informal relations have been identifies as important contributors to learning, especially regarding tacit knowledge [55].

## Methods

Statistical methods for the analysis of social networks have the potential to provide systematic insights into the relational aspects of learning, ultimately opening up the possibility for education programs to better utilise both formal and informal learning relations. Using a social network approach we describe the entire network of learning relations that AHP students recalled at the end of a six-week community-based service-learning program. In particular, our analysis highlights the attractiveness of certain types of people as sources of learning, homophily among students, as well as the reciprocity of learning relations. Acknowledging that the learning outcomes in these programs are diverse, we investigate four domains of learning: clinical knowledge, procedural skills professional development, and the understanding of complex determinants of health.

### Research context

The context for this research study is the “Allied Health in Outback Schools Program” (AHOBSP), an allied health service-learning initiative, that is one of several programs covering students from 16 differing disciplines [56], developed in a fifteen-year collaboration between the community of Broken Hill, the Broken Hill University Department of Rural Health (BHUDRH), several universities, and other key stakeholders including government. Broken Hill is an outer regional population centre of 19,500 in Far West New South Wales, Australia. The region is socioeconomically disadvantaged with a high burden of chronic disease, and increased prevalence of behavioural risk factors such as smoking in pregnancy, obesity, and physical inactivity [57]. These issues are amplified across Indigenous communities in the region. As with other rural and remote centres, the Broken Hill health workforce includes a fly-in-fly-out population and struggles with retention of AHPs. This often results in fragmented and incomplete care. The development of the AHOBSP was driven by the needs of the community who traditionally experienced fragmented access to allied health services, challenges in recruiting and retaining AHPs, and a lack of capacity to place allied health students. The model is described in detail elsewhere [58–60] but the key features, developed after consultation with the literature and several rounds of community engagement, are described in Table 1. Student teams are set up to include a diverse range of backgrounds and experience.

**Table 1:** Features of the Allied Health in Outback Schools Program (AHOBSP) implemented in Broken Hill

Orientation	Based on community need and available evidence
Student Placements	Placement of students in cohorts (4–6 students depending on discipline)
	Placement of students in pairs at each site, including inter-professional pairs (peer support)
	For school based programs, intake of cohorts across each school term (4 cohort intakes annually) and for aged care and disability sectors (4–5 cohort intakes annually)
	Promotion of extended lengths of stay for students (6 weeks plus or extending to a 2nd placement opportunity)
Outcomes	Development of generic learning outcomes relevant to rural and remote practice, i.e. cultural education and primary health care practice
Program Structure	Delivery of structured education across the program period (induction/ orientation linked to placement preparation, inter-professional learning opportunities, clinical discussions and reflection and debrief sessions)
	Continuity of student placement across host sites
	Utilisation of flexible models of clinical and non-clinical supervision (discipline specific clinician, non-discipline specific clinicians, non-discipline non-clinical supervision, peers, on and off site supervision).

For the cohort of AHP students in our study (occupational therapy (OT) and Speech therapy) the program included regular classes and workshops at BHUDRH, and the students’ main role was to conduct lessons in local primary schools and kindergartens to further the children’s lingual and motor development. In teams of two or three students had to plan, prepare, conduct and evaluate classes. Team membership was fixed for each school for the duration of the placement, but different combinations of students teamed up in different schools. School teachers were present during each of their classes. Students lived in shared housing facilities with students of other disciplines during their placement.

In this context, we asked the research question “What are the important social relations of AHP students within a community-based rural clinical placement regarding what and from whom the students learn?”

### Instrument development

The survey instrument with which we collected the social learning networks of students was developed from previous research, reporting the different domains of learning in achieving important learning outcomes by the end of an extended rural clinical placement [41]. These consisted of clinical knowledge, procedural skills, professional development (including professional identity) and the understanding of complex determinants of health (see Additional file 1: Table S1).

Our survey focussed on the students’ attribution of learning from other people. In formulating the questions for these relations, we adapted the social network name generator frequently used in the General Social Survey [61]. We prompted students to provide a list of names of people “with whom you had meaningful interactions and conversations relating to your placement.” In the next step, the survey repeated the list of names that the student had provided along with a check box next to each name, asking students to indicate who contributed to their learning in the given domain. Throughout the survey it was possible to return to the name generator and add additional names. We also asked the students to select a classification of each of their contacts from six categories; supervisor, health professional, BHUDRH administrator, peer, schoolteacher, and client (pupil, parent or caregiver) [62].

### Sampling

An entire cohort of ten students (6 speech therapy and 4 OT students) was invited to participate during the final week of their placement. They had spent 6–8 weeks at BHUDRH. They came from the same university and were in the final year of their undergraduate degrees. Nine were female, one was male.

### Data collection

The online survey (Additional file 1: Table S1) was conducted simultaneously with all students in the BHUDRH computer room. The survey was administered online through REDCap (Research Electronic Data Capture), a secure web application for building and managing online surveys and databases [63]. The students were able to write down an unlimited number of relations. Every person that was mentioned by at least one student was included in the network. Variations of name spellings were matched manually. Classification of roles had to be harmonised between respondents, using a lookup table provided by the BHUDRH. We interpreted the nominations as binary, directed links to create the network.

### Data analysis

The members of the network were grouped into six different types, corresponding to that person’s role in relation to the student. These included academic supervisors, local AHPs, school teachers, clients (comprising pupils and their parents or caregivers), student peers, and BHUDRH administrators. Descriptive analysis summarised how often students reported a learning relationship in each of the four learning domains (ego-centric network analysis). Inductive statistical models quantified the structure of the ten students’ combined network by estimating the relative strength of relational processes that may have contributed to the structure of learning networks. These models are referred to as exponential random graph models (ERGMs). They model the probability of the existence of a tie as a dependent variable in networks where observations are not independent. The investigated relational processes include:

- **Density**: Similar to the intercept in other regression models, this effect is included to represent the overall number of relations observed in a network.
- **Attractiveness specific roles**: Quantifies how attractive different roles are (i.e. academic supervisors, peers, etc.) for students’ learning relations. The estimated coefficients reflect how much more likely a relation to someone in a particular role is relative to a baseline role category. Between students this describes the effect of homophily.
- **Reciprocity among students**: Captures the effect of mutual peer learning relations, i.e. relations where both students indicated that they have learned from the other.

ERGMs allowed us to test if these processes led to significantly more (or fewer) ties than would be expected by chance, i.e. in comparison to random networks for the same group of people. The attractiveness of relations to any of the roles is calculated using ‘local health professionals’ as a baseline. This group was chosen since student experiences should align with what practising health professionals are expected to know, do and value [14, 15]. The model was configured to consider only learning networks that originated from one of our student respondents, as other relations that do not include students were not part of our sample..

ERGMs provide inference statistical tests to assess if these effects are significantly different from zero [64, 65]. Analogous to logistic regression models, ERGM coefficients are interpreted as additive factors on the log odds of forming a learning relationship. Significant and positive coefficients indicate higher log odds of forming a relation in accordance to the underlying process; negative coefficients make it less likely. In the tabular output significant coefficients are highlighted with asterisks that indicate the associated *p*-value. The goodness of fit of each of these models was assessed following established procedures [66].

Analyses were conducted in R version 3.3.0 [67], using the statnet suite of packages (v. 2016.9) [68].

## Results

**Fig. 1:**
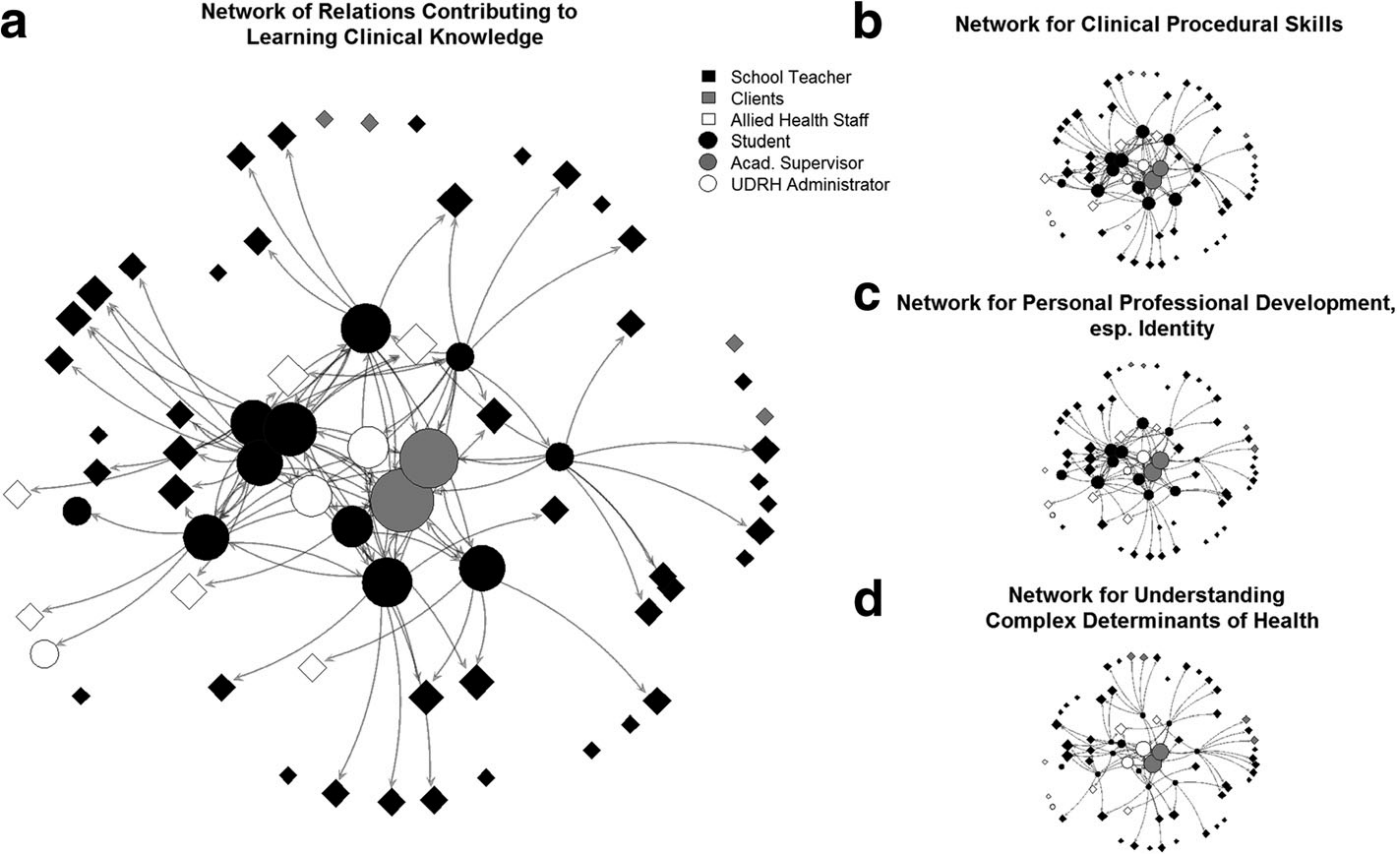
Visualisations of students’ learning networks. People are represented as circles and squares, and shaded by their membership to communities of practice. Learning relations are represented as arrows going out from responding students, pointing to the person they learned from. The visualisations combine the networks of all ten students because in the setting of this placement, different students nominated the same people, as well as their peers. Panel **a**) shows the learning network for clinical knowledge, **b**) for procedural skills, **c**) for professional development, and **d**) for understanding complex determinants of health

Data was available from the networks of the 10 students. Together they reported the presence or absence of a relation to 69 people, providing a data set of 680 ties. The networks of students’ learning relations for each of the four domains are depicted in Fig. 1. A circle or square represents each person that is mentioned by the students. The large panel on the left (a) shows the network of learning relations that were seen as relevant for clinical knowledge. Individual roles are distinguished by colour and shape. The size of individual symbols represents how often a person was named as a learning relation in that particular network.

The smaller graphs on the right-hand side of Fig. 1b, c, d maintain the same positioning of network members but illustrate the networks for the remaining learning domains of procedural skills, professional identity, and complex determinants of health. Visual inspection readily reveals important aspects of the structure of these networks. Across all learning dimensions there are two academic supervisors that are considered important sources of learning by the majority of students. Consistently; these are the biggest symbols. The two networks for clinical knowledge and procedural skills are most densely connected, indicating that students perceived they received valuable input from a wide range of sources. They indicated relevant learning from most of the local role types and from their student peers. The number of learning relations with peers was high in all but the last network for understanding the complex determinants of health. Furthermore, students perceived that clients (pupils and their caregivers) did not contribute to their learning of clinical skills and procedures, and only contributed in a limited way to their professional identity or their understanding of complex determinants of health.

A summary of reported relations from the ego centric analysis is given in Table. 2. The table confirmed the descriptions above. The first three networks were similar in composition, whereas the last one revealed a different pattern of learning: Complex determinants of health showed the fewest average learning relations, students were nominated less, and admin personnel and clients more often. The number reported of relations showed wide variation. Across all learning domains, we found a fourfold difference between the student with the lowest number of relations and the student with the highest number.

**Table 2:** Median number of learning relations reported by the students for each learning dimension (ego-centric analysis)

Role of individual (Total number in the sample)	Clinical Skills: Knowledge	Clinical Skills: Procedures	Professional Identity	Complex Determinants of Health
Acad. Supervisor (2)	2	2	2	2
Local Health Professionals (6)	1	1	1	0
Clients (4)	0	0	0	0
Students (11)	4	4	2	0
Primary School Teachers (43)	3	4	3.5	3.5
BHUDRH Administrator (3)	0	0	0	1
Median Relations per Student (min; max)	11 (4; 17)	10.5 (4; 16)	8 (3; 17)	8.5 (2; 12)

To statistically test if and how these networks differed, we configured one ERGM for each learning domain, including an overall effect for density, relative effects for roles of people, and the effect for reciprocity in peer learning relations. Results are summarised in Table 3.

**Table 3:** Results of ERGM analysis across students (*n* = 10), influential roles (*n* = 6), and alters (*n* = 69)

	Network for Clinical Skills: Knowledge		Network for Clinical Skills: Procedures		Network for Professional Identity		Network for Understanding Complex Determinants of Health	
	Coefficient	Std. Error	Coefficient	Std. Error	Coefficient	Std. Error	Coefficient	Std. Error
*Density*	**− 9796**	1495.9^a^	**−10,355**	1565^a^	**− 8167**	1678.9^a^	**−10,309**	201.9^a^
*Reciprocity among pairs of students*	**1.98**	0.65^b^	**2.41**	0.66^a^	**2.60**	0.73^a^	*not observed*	
*Relations to Academic Supervisors*	**4.30**	0.79^a^	**3.93**	0.72^a^	**4.63**	1.07^a^	**4.39**	0.87^a^
*Relations to other Students (Homophily)*	0.82	0.46	0.74	0.47	−0.11	0.51	**−2.40**	1.09^c^
*Relations to Teachers*	−0.28	0.36	−0.09	0.37	−0.65	0.40	−0.11	0.46
*Relations to BHUDRH Administrators*	0.91	0.54	0.60	0.60	0.13	0.61	**1.35**	0.59^c^
*Relations to Clients*	*not observed*		*not observed*		−1.95	1.09	0.00	0.68

Signif. codes: 0 ^a^0.001 ^b^0.01 ^c^0.05

The models for clinical knowledge, procedural skills and professional identity show the same patterns: Academic supervisors were the only role that contributes to students’ learning significantly more than learning from local allied health practitioners. The formation of learning relations with other roles was no more or less likely than the formation of a learning relation with an allied health practitioner. Learning relations relating to clinical knowledge or procedural skills were not observed with clients at all. At the same time, reciprocity in learning relations amongst pairs of students was a significant effect. This means that it is more likely that student A learned from student B when student B learned from A as well. This effect was significant and made these relations 7.2 (e^1.98^) times more likely around clinical knowledge and 13.5 (e^2.6^) times more likely around professional identity.

In learning relations about complex determinants of health, this reciprocal effect was not observed at all. The reported network contained not a single mutual learning relation between students and therefore the reciprocity term was removed from this model. A further difference in this learning domain compared to the other three is that the coefficient for forming learning relation with peers was negative and significant (e^-2.4^), which means that student peers were 11 times less likely to contribute learning than practitioners about these issues. At the same time, a positive and significant coefficient (e^1.35^) means that local staff at BHUDRH who are not employed in a teaching capacity were 3.8 times more likely to contribute to learning about determinants of health.

Students reported many learning relations with teachers in the schools they visited. While there were not significantly more than by chance in the observed network, there were consistently many in number in all learning domains, as teachers made up the largest group in the network.

## Discussion

This paper adds to the literature about social learning processes in community-based service-learning programs informed and shaped by theoretical frameworks drawn from student work integrated learning, peer assisted learning and social network theory. It demonstrates how the complex interactions facilitated through community-based clinical placements can be analysed systematically to identify significant aspects of the students’ learning network. Allied health students, participating in a community-based service-learning program, reported learning through interactions with supervisors, peers, community members and other personnel with whom they engage in their placements. In all learning dimensions, the academic supervisor was the most likely source of meaningful learning, consistent with the AHOBSP service-learning model. An academic clinician is purposefully employed to be the key educator, responding to challenges associated with recruitment and retention of clinicians within rural health services and to the inability to rely on hospital practitioners as consistent supervisors [59].

Moreover, our data show that students tend to form pairs of reciprocal peer learning relationships where both participants claim to have learned from each other. This effect is evident in the learning domains of clinical knowledge, procedural skills and professional development – all areas where students may be holding some expertise on their own that they are able to share with their peers. This is evidence for a reciprocity effect in this program [50], and may be a reflection in how AHOBSP encourages peer learning by setting up small student teams to visit individual schools, as well as their shared accommodation. Whilst health professional programmes have investigated formal interventions to promote peer learning, and noted the associated cognitive, pedagogical, attitudinal, social and economic benefits [39, 69–71], our finding provide a mechanism of visualising and analysing the peer networks themselves.

However, the lack of reciprocity effect in the domain of complex determinants of health, and the significant, negative coefficient for learning from other students, indicate that peers contributed significantly less to this kind of learning. Students may have limited exposure and experience of the marginalisation associated with the cultural context and socio-economic disadvantage, thus they were rarely able to offer each other relevant insights. At the same time the reported ties to administrative staff revealed another pathway for students to learn about the contextual factors of their work. A potential explanation is that all staff are living in the local community and can provide a broad picture to help students better understand the context of their work, the backgrounds of their clients, as well as the community as a whole.

Our analysis shows that the learning relationships formed by students are established within the formal program, as well as without, confirming the importance of informal, potentially serendipitous relations [43, 44, 55]. These relationships are important in their own right as they may be the source of novel or different information, illustrating “the strength of weak ties” [72].

These insights suggest that workplace learning is a social process, and our detailed quantitative analysis adds a more nuanced understanding of the social processes driving learning. Some groups of people are more frequently seen as sources of learning, reciprocal, as well as informal, relations matter, and patterns differ across learning domains. The wide variation in the number of relations reported by the students, across all learning domains, may be a reflection of variations in student engagement in the program and reinforce the benefits of learner agency [14, 15, 34].

### Implications

For clinical educators and designers of a curriculum, an understanding of students’ learning relations may reveal potential points of leverage to create and improve more targeted education and training strategies for allied health students in non-traditional community-based placements, especially for collaborative education models.

There are a number of implications for health professional educators developing similar community-based service-learning programs that arise from our analysis, regarding program design to enhance social learning, fostering student engagement, supervision, orientation, and peer learning.

The formal curriculum in community-based clinical placement is provided around structured activities organised by both the home university and the program of the hosting department. However, informal social learning may be unstructured and unplanned such as ad-hoc reflections with peers, or serendipitous conversations with administrators [43, 44]. Both formal and informal learning contribute in differing ways to students acquiring appropriate clinical knowledge, skills and competencies, professional identity, and contextual knowledge of the community. This evidence of social learning suggests greater flexibility in considering the sources of learning. It recognizes the significance of learning from other people, but also implies greater scope for individual agency in terms of realising learning opportunities [14, 15, 17, 73], seeing that learning occurs in a much wider variety of settings than formal education or training [43]. Our findings emphasise that this can be a structured part of the program or emerging in the unstructured context of the program, if there are suitable opportunities.

In this study, the supervisor student ratio was 1:6 for speech therapy and 1:4 for occupational therapy students. Given the pressure to provide best practice placements to growing student numbers, this research adds to the limited empirical evidence that placing multiple students with clinical educator may increase capacity facilitated by peer assisted learning [1]. However, in community based placements, the role of the supervisor [74] in the placement needs to evolve to supervising single students to take account of the importance of the broader, unstructured learning environment and the social networks within it. Clinical supervisors could usefully focus student attention on learning strategies which encourage the student’s own participation in it [75]. In this way they could enable and encourage agentic engagement [34] and learning in students [35] through the forming and maintaining of social learning relationships as part of their workplace experience [40, 76]. The value of student learning from and with others could broaden the focus of health professional educators, away from reliance on hierarchical apprenticeship models of clinical education approaches, to distributed approaches that promote social learning. Seeing that reciprocal learning relations emerged from the students’ interactions indicates that there is a collaborative process to learning in this clinical placement, where students learned with each other, and from each other in pairs. In programs where advanced students can be expected to have some degree of expertise, our data shows that reciprocal learning relations can flourish under a collaborative model where one clinical educator supervises two or more students concurrently [3]. A number of studies consistently report overall experience by clinical educators and students in community-based placements [7, 9, 10, 20]. We suggest that future research could adapt the methodology of our social learning network analysis to local contexts. This may provide quantitative measures of the multiple factors underpinning learning in clinical placements and potentially relate them to measures of student learning outcomes.

### Strengths and limitations

A strength of this study is that students not only provided information about whom they had learned from, but also about who they had *not* learned from. Thus our survey provides a census of all of their recalled learning relations at the end of their placements. However, a number of limitations need to be acknowledged. First, this is data from a single community-based rural service-learning program, which limits the generalizability of our findings. Second, we acknowledge that there are problems associated with our approach of collecting relational data through recall [77]. We aimed to minimise the effect of forgetting by conducting this survey while the students were still on placement. Also, our analysis relies on the validity of survey questions and especially how they described the four domains of learning. Given there was not standardised instrument available in the literature, we relied on learning domains validated in previous work [40].

Beyond that our analysis treats learning relations as binary and therefore does not allow us to investigate their quality. Also, our participants were asked to report “meaningful learning relationships”. To do this they had to have insight about their learning process, and it is plausible that tacit learning had occurred, for instance with clients, that they were simply unaware of.

Nonetheless, it is interesting that students in this study consistently do not perceive that they learned from clients or their caregivers. This may reflect broader issues associated with parental engagement within the rural context, structure of the program and duration of the placement.

In comparison to survey studies that focus on the individual, our number of observations may appear low. However, the target of our analysis is the learning *relationship* and its determinants in the context of the students’ network. Our social network survey provided information on several hundred of these relations and therefore establishes the basis for our quantitative analysis. On this basis we then inferred the relevance and magnitude of social processes that are consistent with the presence and absence of relations in our sample.

Lastly, the chosen sampling method restricts our capacity to investigate learning relations that involve more than two people. Social network analysis often investigates the effect of local cliques, emerging through a process of friends connecting with the friends of their friends, so-called triadic closure [53]. However, our sampling method does not capture the full network of relations between every member in the network, as we know only of relations reported by students. This may have reduced the number of local cliques reported in the network and in response we restricted the analysis to networks consistent with this sampling scheme.

## Conclusions

Understanding the structure of student learning relationships through a social network analysis helps identify potential points of leverage to create and improve more targeted education and training strategies for allied health students to optimise the use of community-based service-learning programs. Clinical educators could promote social learning by preparing students to more readily engage with each other and with members of the community.

## Additional file

Additional file 1: **Table S1.** Details of the social network online survey. Questions from social network survey administered online through REDCap (Research Electronic Data Capture). Students nominated peers that contributed to their learning in the areas of clinical knowledge, clinical processes and procedures, professional identity and complex determinants of health. (DOCX 13 kb)

## Abbreviations

AHOBSP: Allied Health in Outback Schools Program
AHP: Allied Health Professional
BHUDRH: Broken Hill University Department of Rural Health
ERGM: Exponential Random Graph Model
OT: Occupational Therapy

## References

[1] Sevenhuysen S, Thorpe J, Molloy E, Keating J (2017). Haines T peer-assisted learning in education of allied health professional students in the clinical setting: a systematic review. J Allied Health.

[2] Lekkas P, Larsen T, Kumar S, Grimmer K, Nyland L, Chipchase L, Jull G, Buttrum P, Carr L, Finch J (2007). No model of clinical education for physiotherapy students is superior to another: a systematic review. Aust J Physiother.

[3] Briffa C, Porter J (2013). A systematic review of the collaborative clinical education model to inform speech-language pathology practice. Int J Speech Lang Pathol.

[4] Secomb J (2008). A systematic review of peer teaching and learning in clinical education. J Clin Nurs.

[5] Currens JB (2003). The 2:1 clinical placement model: review. Physiotherapy.

[6] Jones D, McAllister L, Lyle D. Community-based service-learning: A rural Australian perspective on student and academic outcomes of participation. International Journal of Research on Service-Learning and Community Engagement 2016; 4(1).

[7] Taylor Christine, Angel Liz, Nyanga Lucy, Dickson Cathy (2017). The process and challenges of obtaining and sustaining clinical placements for nursing and allied health students. Journal of Clinical Nursing.

[8] Stewart T, Wubbena ZC (2015). A systematic review of service-learning in medical education: 1998–2012. Teach Learn Med.

[9] Kamien MA (1996). Comparison of medical student experiences in rural Speciality and metropolitan teaching hospital practice. Aust J Rural Health.

[10] Averill NJ, Sallee JM, Robinson JT, McFarlin JM, Montgomery AA, Burkhardt GA, Schulz-Burton MD, Elam CL (2007). A first-year community-based service learning elective: design, implementation, and reflection. Teach Learn Med.

[11] McNeal MS, Buckner AV (2012). using mini-grants and service-learning projects to prepare students to serve underserved populations. J Health Care Poor Underserved.

[12] Stearns JA, Stearns MA, Glasser M, Londo RA (2000). Illinois RMED: a comprehensive program to improve the supply of rural family physicians. Fam Med.

[13] Leung K-K, Liu W-J, Wang W-D, Chen C-Y (2007). Factors affecting students’ evaluation in a community service-learning program. Adv Health Sci Edu.

[14] Billett S (2015). Readiness and learning in health care education. Clin Teach.

[15] Billett Stephen, Sweet Linda (2015). Participatory practices at work: Understanding and appraising healthcare students' learning through workplace experiences. Researching Medical Education.

[16] Lloyd B, Pfeiffer D, Dominish J, Heading G, Schmidt D, McCluskey A (2014). The New South Wales allied health workplace learning study: barriers and enablers to learning in the workplace. BMC Health Serv Res.

[17] Billett S (2001). Learning through work: workplace affordances and individual engagement. J Work Learn.

[18] Manley Kim, Titchen Angie, Hardy Sally (2009). Work-based learning in the context of contemporary health care education and practice: A concept analysis. Practice Development in Health Care.

[19] Buckner AV, Ndjakani YD, Banks B, Blumenthal DS (2010). Using service-learning to teach community health: the Morehouse School of Medicine Community health course. Acad Med.

[20] Burrows MS, Chauvin S, Lazarus CJ, Chehardy P (1999). Required service learning for medical students: program description and student response. Teach Learn Med.

[21] En Wee L, Xin YW, Koh GC-H (2011). Doctors-to-be at the doorstep–comparing service-learning programs in an Asian medical school. Med Teach.

[22] Meili R, Fuller D, Lydiate J (2011). Teaching social accountability by making the links: qualitative evaluation of student experiences in a service-learning project. Med Teach.

[23] Elam CL, Musick DW, Sauer MJ, Skelton J (2002). How we implemented a service-learning elective. Med Teach.

[24] Waddell RF, Davidson RA (2000). The role of the community in educating medical students: initial impressions from a new program. Educ Health.

[25] Roberts C, Daly M, Kumar K, Perkins D, Richards D, Garne D (2012). A longitudinal integrated placement and medical students’ intentions to practise rurally. Med Educ.

[26] Spiers M, Harris M (2015). Challenges to student transition in allied health undergraduate education in the Australian rural and remote context: a synthesis of barriers and enablers. Rural Remote Health.

[27] Ten Cate O, Durning S (2007). Dimensions and psychology of peer teaching in medical education. Med Teach.

[28] Yu TC, Wilson NC, Singh PP, Lemanu DP, Hawken SJ, Hill AG (2011). Medical students-as-teachers: a systematic review of peer-assisted teaching during medical school. Adv Med Educ Pract.

[29] Smith T, Brown L, Cooper R (2009). A multidisciplinary model of rural allied health clinical-academic practice: a case study. J Allied Health.

[30] Burgess A, McGregor D, Mellis C (2014). Medical students as peer tutors: a systematic review. BMC Med Educ.

[31] Budgen C, Gamroth L (2008). An overview of practice education models. Nurse Educ Today.

[32] Perkins GD, Hulme J, Bion JF (2002). Peer led resuscitation training for healthcare students: a randomised controlled study. Intensive Care Med.

[33] Hurley KF, McKay DW, Scott TM, James BM (2003). The supplemental instruction project: peer devised and delivered tutorials. Med Teach.

[34] Reeve J, Tseng C-M (2011). Agency as a fourth aspect of students’ engagement during learning activities. Contemp Educ Psychol.

[35] Billett S (2009). Developing agentic professionals through practice-based pedagogies. Final report for the ALTC Associate Fellowship Retrieved on December.

[36] Thistlethwaite JE (2016). Practice-based learning across and between the health professions: a conceptual exploration of definitions and diversity and their impact on interprofessional education. Int J Pract based Learn Health Soc Care.

[37] Dubé TV, Schinke RJ, Strasser R, Couper I, Lightfoot NE (2015). Transition processes through a longitudinal integrated clerkship: a qualitative study of medical students' experiences. Med Educ.

[38] Yardley S, Teunissen PW, Dornan T (2012). Experiential learning: AMEE guide no. 63. Med Teach.

[39] Jones D, McAllister L, Lyle D (2015). Interprofessional academic service-learning in rural Australia: exploring the impact on allied health student knowledge, skills, and practice. A qualitative study. Int J Pract Based Learn Health Soc Care.

[40] Roberts Chris, Daly Michele, Held Fabian, Lyle David (2016). Social learning in a longitudinal integrated clinical placement. Advances in Health Sciences Education.

[41] Daly M, Perkins D, Kumar K, Roberts C, Moore M (2013). What factors in rural and remote extended clinical placements may contribute to preparedness for practice from the perspective of students and clinicians?. Med Teach.

[42] Teherani A, Irby DM, Loeser H (2013). Outcomes of different clerkship models: longitudinal integrated, hybrid, and block. Acad Med.

[43] Eraut M (2000). Non-formal learning and tacit knowledge in professional work. Br J Educ Psychol.

[44] Hafler JP, Ownby AR, Thompson BM, Fasser CE, Grigsby K, Haidet P, Kahn MJ, Hafferty FW (2011). Decoding the learning environment of medical education: a hidden curriculum perspective for faculty development. Acad Med.

[45] Isba R, Woolf K, Hanneman R (2017). Social network analysis in medical education. Med Educ.

[46] Lusher D, Koskinen J, Robins G (2012). Exponential random graph models for social networks: theory, methods, and applications: Cambridge University press.

[47] Hommes J, Rienties B, de Grave W, Bos G, Schuwirth L, Scherpbier A (2012). Visualising the invisible: a network approach to reveal the informal social side of student learning. Adv Health Sci Edu.

[48] Rienties B, Héliot Y, Jindal-Snape D (2013). Understanding social learning relations of international students in a large classroom using social network analysis. High Educ.

[49] Woolf K, Potts HWW, Patel S, IC MCM (2012). The hidden medical school: a longitudinal study of how social networks form, and how they relate to academic performance. Med Teach.

[50] Blau PM (1964). Exchange and power in social life.

[51] McPherson Miller, Smith-Lovin Lynn, Cook James M (2001). Birds of a Feather: Homophily in Social Networks. Annual Review of Sociology.

[52] Cartwright D, Harary F (1956). Structural balance: a generalization of Heider's theory. Psychol Rev.

[53] Simmel G (1950). The sociology of Georg Simmel.

[54] Granovetter M (1973). The strength of weak ties. Amer J Sociol.

[55] Nie K, Lin S, Ma T, Nakamori Y (2010). Connecting informal networks to management of tacit knowledge. J Syst Sci Syst Eng.

[56] Lyle D, Morris J, Garne D, Jones D, Pitt M, Walker T, Weston R (2006). Value adding through regional coordination of rural placements for all health disciplines: the Broken Hill experience. Aust J Rural Health.

[57] Kennedy C (2005). Health in the Murdi Paaki: Broken Hill Centre for Remote Health Research, University Department of Rural Health.

[58] Jones D, Grant-Thomson D, Bourne E, Lyle D (2011). Investing in the future of rural and remote allied health and kids. 11th National Rural Health Conference*:* 2011.

[59] Jones D, Grant-Thomson D, Bourne E, Clark P, Beck H, Lyle D (2011). Model for rural and remote speech pathology student placements: using non-traditional sites and partnerships. Aust J Rural Health.

[60] Jones D, McAllister L, Lyle D, Brunero C, Webb T, Riley S (2015). Improving health and education outcomes for children in remote communities: a cross-sector and developmental evaluation approach. Int J Community Res Engagement.

[61] Davis JA, Smith TW, Marsden PV (2007). General social surveys, 1972–2006: cumulative codebook.

[62] Marin A (2004). Are respondents more likely to list alters with certain characteristics?: implications for name generator data. Social Netwks.

[63] Harris PA, Taylor R, Thielke R, Payne J, Gonzalez N, Conde JG (2009). A metadata-driven methodology and workflow process for providing translational research informatics support. J Biomed Inf.

[64] Robins G, Pattison P, Kalish Y, Lusher D (2007). An introduction to exponential random graph (p*) models for social networks. Soc Networks.

[65] Morris M, Handcock MS, Hunter DR (2008). Specification of exponential-family random graph models: terms and computational aspects. J Stat Soft.

[66] Hunter DR, Goodreau SM, Handcock MS (2008). Goodness of fit of social network models. J Amer Statistical Assoc.

[67] Team RDC R (2009). A language and environment for statistical computing.

[68] Handcock MS, Hunter DR, Butts CT, Goodreau SM, Morris M (2008). Statnet: software tools for the representation, visualization, analysis and simulation of network data. J Stat Softw.

[69] Topping KJ (1996). The effectiveness of peer tutoring in further and higher education: a typology and review of the literature. High Educ.

[70] Topping KJ (1998). Peer assessment between students in college and university. Rev Educ Res.

[71] Jones D, McAllister L, Lyle D (2015). Stepping out of the shadows: allied health student and academic perceptions of the impact of a service-learning experience on student’s work-readiness and employability. J Teach Learn Grad Employability.

[72] Granovetter M (1983). The strength of weak ties: a network theory revisited. Soc Theory.

[73] Richards J, Sweet L, Billett S (2013). Preparing medical students as agentic learners through enhancing student engagement in clinical education. Asia Pac J Coop Educ.

[74] Kilminster SM, Jolly BC (2000). Effective supervision in clinical practice settings: a literature review. Med Educ.

[75] Collin K, Valleala UM (2005). Interaction among employees: how does learning take place in the social communities of the workplace and how might such learning be supervised?. J Educ Work.

[76] Griffiths T, Guile D (2003). A connective model of learning: the implications for work process knowledge. Eur Educ Res J.

[77] Brewer DD (2000). Forgetting in the recall-based elicitation of personal and social networks. Social Netwks.

