# Supplementary material for: Learning relationships in community-based service-learning: a social network analysis
Source: BMC Med Educ. 2019 Apr 25;19:113. doi: 10.1186/s12909-019-1522-1 (PMC6482529; doi:10.1186/s12909-019-1522-1)
Supplement: Supplementary file 1 — Table S1. Details of the social network online survey. Questions from social network survey administered online through REDCap (Research Electronic Data Capture). Students nominated peers that contributed to their learning in the areas of clinical knowledge, clinical processes and procedures, professional identity and complex determinants of health. (DOCX 13 kb) [file 12909_2019_1522_MOESM1_ESM.docx]

| **Name Generator** |
| --- |
| Think about the people that you have met during the last weeks of placement in Broken Hill. To help us understand who contributed to your learning experience, please provide the names (first and family names) of those people with whom you had meaningful interactions and conversations relating to your placement. These conversations could mean sharing of factual knowledge and information, discussing cases, trying to solve particular problems, planning your sessions, exchanging tips regarding how to deal with pupils or other people, developing a plan, action, or other activity together, and/or learning about the broader circumstances and context of people here.  Also, for each of the people that you list here, please select from the drop-down menu what kind of role they have in relation to you. |
| **Drop-down menu for roles (aggregated for analysis)** |
| Administrator (School); Administrator (BHUDRH); Other Health Professional; Principal; Pupil; Pupil's Parent; Pupil's Caregiver; Student (medical); Student (allied-health); Student (other); Supervisor (discipline-specific); Supervisor (non-discipline specific); Teacher; other |
| **Clinical Skills: Clinical Knowledge** |
| Please identify by ticking the box next to their name, all the people that provided you with clinical knowledge and/or factual information relevant to your learning during your placement. |
| **Clinical Skills: Clinical Processes and Procedures** |
| Please identify by ticking the box next to their name, all the people that provided you with generic, practical or procedural assistance related to your service. This can include people who helped you plan your sessions, deal with particular issues about your cases, as well as people who collaborated with you or who gave you hands-on practical support. |
| **Personal Professional Development: Professional Identity** |
| Please identify by ticking the box next to their name, all the people that affected you in the way you think about the role of allied health in society, and your profession in general. |
| **Complex Determinants of Health** |
| Please identify by ticking the box next to their name all the people that helped you better understand the broader context of health in rural and remote communities, including the cultural, social and economic aspects. |
